# Digital well-being under pandemic conditions: catalysing a theory of online flourishing

**DOI:** 10.1007/s10676-021-09584-0

**Published:** 2021-03-01

**Authors:** Matthew J. Dennis

**Affiliations:** Technische Universiteit Delft, Delft, Netherlands

**Keywords:** Digital well-being, COVID-19, Pandemics, Human flourishing

## Abstract

The COVID-19 pandemic has catalysed what may soon become a permanent digital transition in the domains of work, education, medicine, and leisure. This transition has also precipitated a spike in concern regarding our digital well-being. Prominent lobbying groups, such as the Center for Humane Technology (CHT), have responded to this concern. In April 2020, the CHT has offered a set of ‘Digital Well-Being Guidelines during the COVID-19 Pandemic.’ These guidelines offer a rule-based approach to digital well-being, one which aims to mitigate the effects of moving much of our lives online. The CHT’s guidelines follow much recent interest in digital well-being in the last decade. Ethicists of technology have recently argued that character-based strategies and redesigning of online architecture have the potential to promote the digital well-being of online technology users. In this article, I evaluate (1) the CHT’s rule-based approach, comparing it with (2) character-based strategies and (3) approaches to redesigning online architecture. I argue that all these approaches have some merit, but that each needs to contribute to an integrated approach to digital well-being in order to surmount the challenges of a post-COVID world in which we may well spend much of our lives online.

## Emerging threats to digital well-being

In April 2020, YouTube’s CEO, Susan Wojcicki, predicted that the pandemic would cause ‘an acceleration of our digital lives’ (Stelter and Wojcicki 2020). Wojcicki could have put her claim more strongly. At the time of writing (2020), the pandemic has caused millions to integrate digital technologies into their daily routines at an unprecedented rate. Within a very short time period, entire populations transformed their lives, and started working, socialising, shopping, and seeking medical attention online. Indeed, the pandemic has not only transformed personal lives, it has changed how we interact collectively. It has transformed social and institutional working practices in ways that look increasingly likely to be retained after SARS-CoV-2 has been vanquished. Steve Petruk, chief operating officer of a global outsourcing firm, notes that COVID-19 has incentivised companies to fast-track their plans for digital transition in a matter of days or weeks, a process they had previously anticipated taking decades. Now his company’s transition is complete, Petruk tells us, the new working practices that the pandemic has precipitated ‘will define a new normal for years to come’ (McIntosh and Petruk 2020).^1^

While COVID-19 is catalysing change in the commercial sphere, digital transformation is also happening at an institutional and governmental level. On 5th May 2020, the governor of New York, Andrew Cuomo, announced plans for a partnership with the Bill and Melinda Gates Foundation, which would aim to ‘reimagine education’ by creating ‘virtual classrooms’ (Strauss 2020).^2^ Soon afterwards, Eric Schmidt (Alphabet CEO 2015–17, Google CEO 2011–15) endorsed Gov. Cuomo’s plans, prophesising that the effects of COVID-19 will now allow technology corporations to digitalise key public services that had previously been resistant to private partnership (Klein 2020). For Schmidt, the pandemic creates innumerable opportunities to ‘use technology to make things better’, including in ‘telehealth’, ‘remote learning’, and ‘social activities.’^3^ While big tech’s plans to rollout wholesale online services at a societal level were gathering pace before the SARS-CoV-2 virus struck, Schmidt’s prediction that the pandemic will hasten this process now seems all but certain.^4^

Whether caused by climate change or another pandemic, the consequences of such a large-scale shift in our online behaviour is still unpredictable. Nevertheless, there is no doubt that spending extended periods online will affect our digital well-being.^5^ The precise effects of long-term online usage are still contested in the psychological literature (Goh et al. 2019; Goodyear et al. 2018; Orben and Przybylski 2019a, 2019b. Cf. Samad et al. 2019; Twenge 2020), but – aside from debates about the dangers (or otherwise) of screentime – there is a consensus that our ability to flourish is impacted upon by our online behaviour (Burr and Floridi 2020; Burr et al. 2018, 2020; Dennis 2020a, 2020b; Vallor 2012, 2016). How we are affected by spending most of our waking lives online is neither solely a medical question (how screentime affects eyesight, say), nor simply a psychological one (how being online continuously affects mental health). Instead, it is one that requires us to ask more broadly what role online technologies should play in a flourishing human life (Goodyear et al. 2018; Orben et al. 2019b). In sum, this means that, while the pandemic has given the ongoing digital transition a new rationale, it also gives new urgency to the question of how to cultivate our digital well-being.

The aim of this article is to offer a sketch of the various approaches to improving digital well-being during and after the COVID-19, especially if the pandemic permanently increases the amount of time we are required to spend online. To do this, in Sect. Rule-Based Strategies, I begin by exploring the reasons to be cautious of one initiative that has recently been offered by a influential US-based lobbying group, the Center for Humane Technology (CHT). While this initiative is certainly a step in the right direction, I argue that the struggle for digital well-being in the post-COVID world needs to enlist more practical tools, including from the character-based approaches (Sect.Character-Based Strategies) and design approaches to online architecture (Sect. Redesigning Online Architecture for Digital Well-Being). Each of these strategies has distinctive benefits that will be essential to make use of to cultivate digital well-being effectively. I conclude by sketching how these strategies could be integrated in ways that would improve our digital well-being in the post-COVID world.

## Cultivating digital well-being in a pandemic

From the early 2010s, the negative effects of being continuously online gained increasing attention in the mainstream press.^6^ Two longitudinal studies by the Copenhagen-based Happiness Research Institute (HRI) empirically supported ongoing journalistic and anecdotal reports. The first, published in 2015, caused much stir among social media companies as it claimed to find a demonstrable link between the time spent online and life dissatisfaction. The second HRI study, published months before the pandemic struck, presents a more nuanced view, stressing how digital well-being is affected by the ‘quality’ of users’ online activity. Instead of measuring how long users spent on social media, this study focused on how users behave on it, especially on whether they were ‘actively posting’ or ‘passively scrolling’ (Birkjær and Kaats 2019). The authors of this new study concluded that the *way that users engage* with social media affects their digital well-being, rather than *how long* they spent online.^7^ Since the pandemic, the HRI has begun a new study on how the secondary effects of the pandemic (lockdowns, homeworking, extended time spent video-calling) will affect digital well-being (Wiking 2020). Although the results of this study will not appear until summer 2021, existing research gives us reason to be cautious (cited above: Goh et al. 2019; Goodyear et al. 2018; Orben and Przybylski 2019a, 2019b. Cf. Samad et al. 2019; Twenge 2020). This literature suggests there our digital well-being undergoes discerptible changes when we are online for extended periods. Combined with the exigencies of the COVID-19 crisis, existing research on digital well-being indicates that we will need practical strategies for cultivating a healthy online life, especially if the pandemic causes a digital transition in previously offline activities. These practical strategies can be divided into two categories: those that attempt to change users’ ‘extrinsic motivation’ and those that attempt to change their ‘intrinsic motivation’ (Peters et al. 2018, Ryan & Deci, 2017). In what follows, I suggest that ‘rule-based strategies’ and the ‘design of online architecture’ are approaches to digital well-being that aim to change the extrinsic motivation of users; whereas ‘character-based’ strategies aim to affect their intrinsic motivation.^8^ In Sect.A Comprehensive Theory of Digital Well-Being for a Post-COVID World, I conclude that all three strategies will be necessary for cultivating digital well-being in a post-COVID world.

Before examining these strategies in detail, it will be useful to summarise the ethical issues that each involves. On the one hand, changing the intrinsic motivation of users has some ethical advantages over changing their motivation extrinsically. For example, there are times when changing extrinsic motivation amounts to manipulation (introducing a persuasive technology into an online environment, say), whereas changing a user’s intrinsic motivation weakens this charge (such as when we acquire a character trait that we reflectively endorse). On the other, overestimating the power of our intrinsic motivation leaves us vulnerable to being blamed for our online conduct. Recognising that our online habits are deeply guided by persuasive technologies (PTs) that keep us engaged with online platforms, should caution us against attributing too much responsibility to users. PTs are overtly designed to keep us hooked online, I will argue that strategies of extrinsic motivation (such as the CHT’s guidelines for digital well-being) are in danger of burdening users with too much personal responsibility for their online conduct.

As we shall see, both extrinsic and intrinsic motivation strategies court various ethical risks and rewards. Despite my criticisms of strategies of extrinsic motivation, I suggest in Sect. Redesigning Online Architecture for Digital Well-Being that the design of online architecture (an extrinsic approach) can usefully promote digital well-being, while avoiding overburdening us with too much blame and culpability for how we act online. In fact, the design of online architecture involves another kind of ethical risk. It courts charges of paternalism, because this strategy could be said to undermine the autonomy of users. Nevertheless, in general, I conclude that shifting the burden of responsibility for digital well-being from *users* to the *designers* of online platforms is a welcome change. I finish by proposing that the ethical risks and rewards can be best met by adopting an integrated approach. Such an approach seeks the resources to show how we can cultivate digital well-being under pandemic conditions by borrowing from all the strategies outlined in Sects.Rule-Based Strategies,Character-Based Strategies, and Redesigning Online Architecture for Digital Well-Being.

## Rule-based strategies

As the consequences of COVID-19 became apparent, CHT issued a set of directives for the cultivation of digital well-being in April 2020. Here, CHT acknowledges that ‘during the COVID-19 crisis, it is natural for our tech time to increase’, so we need to be especially careful about how we use online products and services. In line with its mandate, the CHT cautions users that ‘many [popular social media] products are actually not on our side’ (2020). It claims that these products can be responsible for ‘addiction,’ ‘self-obsession,’ ‘misinformation,’ and ‘content that outrages and polarizes.’ To combat these problems, CHT sets out eight *rules* that it recommends users follow to combat the pernicious effects of online products (‘Digital Well-Being Guidelines during the COVID-19 Pandemic’).^9^ Each of these rules can be grouped into three categories: (1) abstinence, (2) scepticism, (3) mindfulness and embodiment. As I argue below, the problem with this approach is that countering PTs using a traditional rule-based strategy radically underestimates their power.

### Abstinence

The chief weapon that CHT promotes to safeguard the digital well-being of users is abstaining from online technologies, or at least strenuously self-regulating our use of them. As I have argued elsewhere, abstinence is the cornerstone of most rule-based approaches to technology use (name to be supplied after review). It is part of a long tradition of practices to limit our relationship with digital technology, including pocket-sized Faraday cages, targeted self-help books (Chatfield 2012; de Botton 2016), and most recently the screen time tracking functions of Android’s Pie 9 and Apple’s iOS 12. In this tradition, the Center’s guidelines acknowledge that ‘tech is not neutral. It is vying for our attention and is very good at grabbing and holding it’ (2020). To remedy this, CHT suggests that users ‘consider making a time management plan at the beginning of each day, week, or month.’ Armed with such a plan, the Center hopes, users will be better able to moderate their use of technology, only using it when necessary. An analogy with another addictive product with detrimental effects illustrates this. Take, for instance, fast-food. Both governmental regulators and the fast-food industry itself suggest that burgers and other deep-fried foods can be safely consumed if done so in the context of a healthy lifestyle (exercise, other foods, etc.). In an analogous way, the CHT’s offers users a ‘time management plan’, so that users can limit their time online. This aims to ensure that the lives of users include a healthy balance of digital and a non-digital activities.

Nevertheless, as mentioned above, the problem with this approach is that it drastically underestimates the strength of persuasive technologies (PTs) that technology platforms use to engage their users. Although CHT’s guidance acknowledges this strength (tech ‘is very good at grabbing [our attention] and holding it’), there is copious evidence from the persuasive technology literature that a rule-based approach involving periods of abstinence would be insufficient to counter the effects of the kind of PTs that keep us clicking and scrolling online (Frank 2020; Ijsselsteijn et al. 2006; Lanzig 2018). Again, the fast-food analogy points to a helpful way to think about this. While fast-food retailers may seek to absolve themselves of responsibility for the over consumption of their food (stressing the ‘balanced diet’ argument, etc.), they simultaneously use insidious marketing techniques to target those who they know are not eating their food, encouraging them to consume it as part of a healthy lifestyle. While I conclude in Sect. A Comprehensive Theory of Digital Well-Being for a Post-COVID World that an abstinence-based approach has some role to play in the regulation of pernicious digital habits (as well as dietary ones), we should be cautious about overestimating the effect such a strategy can have. *Underestimating* the power of PTs, leads us to *overestimate* the effectiveness of an abstinence-based approach.

### Scepticism

Both the CHT’s fourth and eighth rules advocate adopting a sceptical attitude to online products and services. The fourth rule alerts users to implicit value ‘trade-offs’, pointing out that much tech use concerns trading immediate ‘convenience’ for ‘social connectivity’.^10^ The final rule reminds users that the business model of companies that provide ‘free social media products’ is to try to ‘get you hooked on sharing information about yourself’ (2020). This model, the CHT cautions us, allows companies to make:

[B]illions of dollars by analysing your data and your behaviour with powerful supercomputers, selling those insights to advertisers who want to sell products to you and your friends. The advertisers are the real customer, and unfortunately, you are the product being sold to them. Remind yourself and your kids of this.

While increasing awareness of how users’ data are used is a valuable thing to do, we should not assume that a warning will be enough to change the behaviour of users. The power that social media companies hold over the online lives of users means that many of us will still want (or need) to use their services, despite the fact that we may know about disreputable data harvesting practices.

### Mindfulness and embodiment

The final CHT rule aims to encourage what they term an ‘embodied’ approach to the use of digital technology under pandemic conditions, as well as promoting a general attitude of ‘mindfulness’ when online. The first rule encourages users to attend to ‘how technology makes us feel’ and to ‘take the time to reflect on how it’s working or not working with your well-being’ (2020). Encouraging users to focus on their body is intended as an antidote to some of the PTs mentioned above. As we will see below, these technologies work by hijacking our cognitive abilities in ways that are capable of controlling our online behaviour. Asking users to return their attention to their bodies is intended to precipitated a more self-reflective attitude towards technology use. To do this, CHT recommends trying to identify:

What thought, feeling, or impulse led you to pick up your device? As you scroll through your feed, what kind of thoughts come up? What kind of emotions come up? What happens to your breathing? How does your heart feel?

Similarly to the first rule’s emphasis on self-questioning, this rule requires that users interrogate themselves while using social media, but in this case to do so in a way that promotes mindfulness. To do this, it presents users with series of questions to ask when they are engaging with online technology, aiming to ensure that our mobile devices are used as ‘tools instead of end in themselves’ (2020). Questions include:

Why am I reaching for my device? How is this technology really enhancing my life? Is this technology serving as a successful substitute for something lacking during the pandemic (i.e. exercise or education)?

Asking these questions, CHT suggests, promotes a more thoughtful attitude towards social media technology. Echoing the HRI’s 2019 study, cited above, the CHT urges us ‘consider the type of activity you […] are doing on screens’, noting that ‘creating or being in conversation is better for well-being than passively scrolling or consuming the content of others’ (2020).

## Character-based strategies

As mentioned in Sect.Cultivating Digital Well-Being in a Pandemic, the problem with rule-based strategies is that they underestimate the power of the PTs to keep users hooked online. Even advocates of rule-based strategies admit this. We saw above that the CHT acknowledges that social media companies are ‘vying for our attention, and are very good at grabbing and holding it’ (2020). Nevertheless, when it comes to issuing guidelines about how to improve digital well-being, the CHT proposes combatting PTs in ways that assume users have a high degree of autonomy^11^ and personal responsibility. The literature on PTs strongly indicates otherwise (Birkjær and Kaats 2019; Frank 2020; Hamari 2014; Lanzig 2018, 2019; Orben 2019, 2020). PTs are highly effective at undermining our autonomy – they are explicitly designed to do this, so it is not surprising that obeying self-imposed rules to cultivate digital well-being often proves to be impossible. This means that, while providing users with guidelines might be useful, if PTs are as effective at keeping us hooked online as the scholarship suggests, then rule-based guidelines can only be a first step towards digital well-being.

One alternative to rule-based approaches are character-based strategies. Instead of offering extrinsic guidelines to combat PTs (presupposing high degrees of autonomy and individual volition), character-based strategies show how users can cultivate character traits – intrinsic qualities – that are conducive to their digital well-being. Recently, some theorists have argued that character-based strategies might be the key to regulating online behavior. As Guy Fletcher puts it, character-based theories propose that ‘we can equip ourselves with powers and capacities to mitigate the attention-hogging effects of digital technologies by developing *specific virtues of attention and the like’* (Fletcher 2020, p. 6; emphasis added). Advocates of character-based theories have even elaborated on what the ‘specific virtues’ for digital well-being might be. Shannon Vallor suggests that we need to regulate our online behaviour by employing ‘techno-moral virtues’ (2016), whereas Tom Harrison offers his own account of ‘cyber virtues’ (Harrison 2016; cf. Dennis and Harrison 2020), which explains how children and young people can live better online lives. In other words, Vallor and Harrison each propose virtues that they think we need to flourish in an online environment, as well as modifications of existing ones. Nevertheless, their approach is similar insofar as they view character traits as an intrinsic source of motivation that can control online behaviour.

A practical extension of character-based theories can be found in self-care app technology. At the outset of the COVID-19 pandemic, many self-care app companies were quick to respond to the fact that potential users would soon be spending extended amounts of time online. Many offered free introductions to their products, initially to those in the medical professions (Headspace: March 2020), but quickly expanded to those in lockdown or quarantine situations more generally (Calm, Aloe Bud, Aura: April 2020). Many of these technologies explicitly repurpose the PTs that social media companies use to keep us hooked online, and aim to redirect them to cultivate digital well-being. Gamification provides an instructive example of how self-care apps aim to repurpose PTs.^12^ Gamification has been shown to be incredibly effective at keeping users continuously engaging with platforms because it eschews users’ executive functions (Goebl et al. 2019, Hamari 2014). Self-care apps such as Happify, an industry leading self-care app, employ gamification to keep users engaged, to monitor their online use, and to ask them to reflect on the emotions that extended periods of online use has generated in them (Parks et al. 2021, Parks 2018). Users are presented with a game-like interface with cartoon depictions of various emotions, and are asked to identify the emotions they are experiencing by touching the screen of their digital device to gain points. It is perhaps no accident that the founders of Happify began their careers in the games industry before embarking on their project – to use their words – to ‘gamify happiness’ (Belli 2016, p. 98).

Thinking ahead to after the pandemic has passed, it may be that gamification can retain a role in guiding the conduct of users – especially children and young people – towards socially responsible online behavior. As we have seen, taking the compulsive power of a PT such as gamification seriously, should cause us to question rule-based strategies that require much willpower and a high degree of personal autonomy from users. PTs are effective because they are able to target users’ psychological weak spots. They have been explicitly designed to evade users’ executive functions, so it is not surprising that self-care companies have viewed the idea of repurposing PTs to cultivate digital well-being has holding promise.

Nevertheless, there are problems with this approach. Two objections are especially difficult to dislodge. We encountered a version of the first objection in above. This concerns the *onus of responsibility*. Offering users rule-based guidelines to guide online behaviour is wanting because it invests users with too much personal responsibility when they (inevitably) break the rules. As we saw, doing this underestimates the power of PTs, as these technologies have been explicitly designed to circumvent our autonomy so they can guide our online behaviour. Nevertheless, this problem is not restricted to rule-based approaches, as it applies to character-based one’s too. Just as we are can be held responsible for ‘breaking the rules’, we can be responsible for our character traits. While we are responsible for our character in a different way to how we are responsible for breaking the rules, personal responsibility exists in both cases; we can be blamed for what we do, but under certain conditions we can also be blamed for who we are. The problem is that both strategies offer an individualised approach to digital well-being, which provides the conditions for blame when we err.

The second objection is connected to the first insofar as it concerns the power of PTs to influence online behaviour. As the first objection makes clear, both rule-based and character-based approaches leave us vulnerable to blame when we err online (digital distraction, obsessive scrolling, etc.) because they conceive digital well-being as something that applies at the level of the individual. Nevertheless, much literature indicates that both our ability to follow the rules, and to act on virtuous character traits, is extremely brittle in the face of environmental cues. To understand why rule-based and character-based approaches need supplementing to form a comprehensive approach to digital well-being involves understanding how powerful digital environments are in shaping online conduct. As we shall see in the next section, redesigning online architecture offers the possibility of shaping our behaviour and a non-individualised level (responding to the first objection), whereas it also takes into account the importance of environmental cues that affect how we behave online.

## Redesigning online architecture for digital well-being

We have seen that rule-based approaches, an extrinsic motivation strategy, burdens the user with a high level of personal responsibility for their digital well-being. Given the effectiveness of PTs in online environments, this presents users with an almost impossible task. The empirical evidence consistently shows that human willpower cannot fully resist the PTs that cause chronic distraction and online addiction (Frank 2020; Lanzig 2019, 2018; Sullivan and Reiner 2019). This means that we would be unfairly blamed for lapses in this area because our individual defences against PTs are easily surmountable.^13^ Both rule- and character-based strategies view digital well-being as a task for individuals, who can be blamed when they fail in this regard. This leaves us with a practical gap in the project of cultivating digital well-being, especially at a time when we are required to spend much of our time online. As I argue in the next section, although personal responsibility has a role to play in cultivating digital well-being, it needs to be complemented with an approach that addresses the environmental cues that guide individual behaviour.

One reason for seeking such a third approach is worry that individualised digital well-being strategies are simply not powerful enough to cultivate digital well-being in the face of the PTs that dominate today’s online environments.^14^ The seeds of this concern can be traced to the so-called ‘situationism debate’, in which personal willpower and character traits were viewed as supervening on environmental cues. Situationists, such as Gilbert Harman, argue that that there is ‘no empirical basis for the existence of character traits’ (Harman 1999, p. 1; cited by Upton, 2009, p. 108). In a similar vein, John Doris uses empirical studies to undermine the widely held belief that character traits can reliably predict behaviour compared to how behaviour could be predicted by environmental cues (2002).^15^ Virtuous moral behaviour, Harman and Doris suggested, is best promoted by redesigning the environments that we live in, rather than requiring us to follow rules or trying to cultivate virtuous character traits.

While most situationist literature has focused on offline environment influences, ethicists of technology have sought to understand a comparable phenomenon in online environments. In *Evil Online*, Cocking and van den Hoven show how there are specific ‘features of our online worlds that erode empathy and moral character’ (2018, p. 4).^16^ Such features, they argue, are geared towards ‘stifling moral and prosocial development’ (2018, p. 4), which gives rise to what they term ‘moral fog’ (2018: Ch. 4). Updating the situationist account of Doris and Harman to account for moral conduct in online environments, they note that:

A good deal of recent empirical research has shown the ways in which the design of the technology, the mechanisms, circumstances, imperceptible sensory cues, and the design of choice situations are hugely important for the way people behave online (2018, p. 5).^17^

For Cocking and van den Hoven, then, online environments are loaded with manipulative e-choice architecture and other PTs that strongly influence how we conduct ourselves online. The problem with both rule-based and character-based approaches is that they underestimate how online environments have been explicitly designed to promote behaviours that are incompatible with digital well-being. The moral fog of these online environments requires us to revise how we evaluate the behaviour of those who use them. It should change how we attribute blame and personal responsibility, but most importantly it should motivate us to rethink how online architecture is designed.

If Cocking and van den Hoven are right in thinking that online environments corrode robust character traits, such as those that promote digital well-being, then character-based strategies such as those proposed by Harris (2016) and Vallor (2012, 2016) have limits. If our characters cannot ensure digital well-being in the face of PTs such as hypernudges (Lanzig 2019), e-choice architecture (Frank 2020), dark patterns (Narayanan et al. 2020), or gamification, then redesigning digital environments may offer a better way to cultivate digital well-being. Fortunately, the idea that we can design online architecture in ways that would be conducive to digital well-being has precedent. Much of this work comes from value-sensitive design (VSD) (van den Hoven 2015). Although the bulk of VSD scholarship in this area concerns how to design for well-being in general, some insights can be applied to designing online architecture for *digital* well-being specifically. This literature gives some important clues to how redesigning online architecture may well have an important role to play in cultivating digital well-being under pandemic conditions.

Writing in 2015, Philip Brey concedes that, although well-being is widely recognised as ‘one of our highest values’, designing for it is ‘still in its infancy’ (2015, pp. 379–80). Nevertheless, Brey argues, there is fertile conceptual ground for such a project because ‘increased well-being is a possible consequence of the use of a technological artefact, [so] it is possible, in principle, to design for well-being’ using the VSD approach. Brey identifies four distinct ways to design for values (1) ‘emotional design’ (2015, pp. 372–4); (2) ‘capability design’; (2015, pp. 374–6); (3) ‘positive psychology approaches’ (2015, pp. 376–7); and ‘life-based design’ (2015, pp. 377–8). Although Brey finds problems with all these approaches, he concurs with an earlier observation by Ibo van de Poel that designing for well-being using VSD is possible in principle. For van de Poel, the project of designing for well-being is possible, if it can surmount what he terms the ‘epistemic’ and ‘aggregation’ problems.^18^ Both these problems may, of course, beset an analogous approach to digital well-being, but there is reason to think that VSD theorists might be in a better position to understand their implications in the digital domain. Regarding the epistemic problem, because digital well-being is a narrower than well-being in general, it may be easier to discern how online use affects users and non-users alike. Similarly, in the case of the aggregation problem, the metrics and data that online behaviour generates may be better able to resolve conflicts and value trade-offs between the digital well-being of groups of users because they are more readily quantified. If the epistemic and aggregation problems can be solved, then a VSD approach seems to have the potential to change how we design online architecture for digital well-being specifically.

Before moving to discuss what a comprehensive theory of digital well-being could look like, it is important to note that a VSD approach to digital well-being has already been practically experimented with in the domain of ‘Positive Technologies’ (Peters et al. 2018, p. 2), a term collectively used to describe ‘Experience Design (Hassenzahl 2010), Positive Design (Desmet and Pohlmeyer 2013a), and Positive Computing (Calvo and Peters 2013, 2014). This work views well-being as a key value for which we can design. Pieter Desmet and Anna Pohlmeyer, for example, argue that there is much scope for designers to pay closer attention to the ‘effects of design on the subjective well-being of individuals and communities.’ (2013b, p. 6) More recently, Dorian Peters et al. have proposed that online architecture could be designed with digital well-being in mind. Claiming to base their model on ‘four decades of empirical research’, they propose that designers could use what they call the METUX model (‘A model for Motivation, Engagement, and Thriving in the User Experience’) (Peters et al. 2018, p. 4). Combined with Brey’s and van de Poel’s optimism about surmounting the epistemic and aggregation problems that beset any project of explicitly designing for well-being, these initiatives further support the idea that designing online architecture for digital well-being is possible.

## A comprehensive theory of digital well-being for a post-COVID world

COVID-19 challenges us to rethink how we cultivate digital well-being. The pandemic requires an urgent response in this regard because lockdowns have required unprecedented numbers of people to simultaneously shift their work and leisure activities online. Nevertheless, the problem of how to live well with online technologies has tracked the rise of these technologies for decades, so the challenge we now face is both old and new. We have become increasingly used to socialising, working, and performing everyday tasks online, which has been accentuated by the ease with which online technologies allows us to do this. These decade-long changes have motivated a variety of interested parties to propose ways to cultivate digital well-being from three main perspectives. As we have seen, NGOs such as the CHT propose a rules-based approach; ethicists of technology such as Vallor and Harris advocate rethinking the character traits we need to thrive online; theorists from the VSD community have proposed conceptual frameworks that can inform our future design of online architecture. My contention is that the challenges of a post-COVID world will require that we make use of all these strategies by integrating them into a comprehensive theory of digital well-being.

Looking ahead to when the COVID-19 crisis has abated, there also seems to be strong prudential reasons to view digital well-being as an urgent and ongoing task. These reasons concern future challenges, either new viruses or future ecological hardships, either of which may require us to collectively become much better at flourishing online. Equipping ourselves with an online infrastructure makes us more resilient to future hardships. But this only make sense if this infrastructure is designed so that it allows us to flourish while spending extended amounts of time online. Vallor sketches a vivid picture of such potentially devastating future hardships when she writes:

Glaring lapses in collective and individual practical judgment have led to widespread and growing environmental degradation and resource depletion, global economic and climate instability, and an increasingly chaotic and violent geopolitics, all of which point to the fragility of human flourishing in our present moral condition (2016, p. 117).

While online technology cannot resolve these problems, it may go some way to mitigating them. COVID-19 caused extended lockdowns that led to renewed interest in remote working, telemedicine, and online events. It has been widely noted that the pandemic has caused a massive reduction in air travel, one which shocked environmentalists who had been lobbying governments and cajoling consumers to do this for years (*The Economist* 2020). In addition to lockdown and quarantine strategies, collective action may be necessary, including thinking of how we can restructure our societies in ways that make them more resilient to pandemics (and other ecological threats). As we have seen, COVID-19 has already precipitated such moves; corporations such as Alphabet, and its subsidiaries, are already starting to offer digital services that they claim can replace flesh-and-blood employees (e.g. teachers) or brick-and-mortar institutions (e.g. classrooms).

Nevertheless, the advantages of using online technologies can only be harnessed if we are clearsighted about the dangers of online activity, if we can mitigate the risks, and most importantly if we can *rethink digital well-being* in imaginative new ways. As we have seen, current initiatives offer theoretical and practical strategies for doing this, but alone each of these strategies is not comprehensive because each contains weaknesses. One way to solve this problem is to think of these strategies as having the potential to play a role in a comprehensive approach to digital well-being. Such a comprehensive approach would use aspects of each existing digital well-being strategy, bolstering the weaknesses of one strategy with the strengths of the others and vice versa. The aim, then, would be to construct a comprehensive approach by combining the various strategies that aim to cultivate digital well-being. What might such a comprehensive approach look like? To sketch such an approach, we can discuss the strategies evaluated in Sects. Rule-Based Strategies,Character-Based Strategies, and Redesigning Online Architecture for Digital Well-Being in reverse order.

First, we saw that an important benefit of redesigning online architecture is that it removes the onus of responsibility for digital well-being from the user, redirecting it towards the designers and providers of online architecture. In my discussion of situationism, I noted that character-based ethicists can overestimate the role of mundane environmental factors, which have often proved to be better predictors of an individual’s moral conduct than their purported character traits. In online environments, I followed Cocking and van den Hoven’s claim users are faced with cues that redirect them, including the PTs that other theorists have shown to be so powerful (Frank 2020; Lanzig 2019, 2018; Sullivan and Reiner 2019). These theorists concur that PTs are extremely effective at undermining self-determination and autonomy, as well as concurring with situationists such as Doris and Harman that concepts such as ‘willpower’ and ‘character’ have limited explanatory value. VSD theorists and Positive Designers adopt a more constructive approach. In their view, it is right that our moral conduct is largely determined by environment, we can still make reflective choices on how we design our environments. Furthermore, VSD theorists such as Brey and van de Poel make conceptual space for designing online environments that prioritise the value of digital well-being. Given the power of these environments to shape our conduct online, this value should be prioritised by those who design and provide online services.

Second, we should take seriously the potential charges of paternalism that might be directed towards an approach to digital well-being that only uses a strategy of extrinsic motivation. While redesigning online environments is an effective way to improve digital well-being, we should be wary that it takes away our ability to choose. As we have seen, today’s PTs are extremely effective at guiding our online behaviour, but it could be argued that, even if these PTs are repurposed for digital well-being, they undermine elements of our autonomy and self-determination. Benign paternalism is still paternalism. This problem is compounded if one thinks that autonomy is an essential dimension of well-being in general and digital well-being in particular, as this means that we need to retain the possibility of morally straying in our design of online architecture. Designing online architecture in a way that balances the autonomy requirement with requirements pertaining to digital well-being would be a difficult task, but the potential rewards are important, as I suggest in the two final points below.

Third, although the literature on PTs emphasises our passivity in the face of environmental cues, there is some merit in retaining with the concepts of ‘willpower’ or ‘character’, at least to some extent. While we should not underestimate the power of PTs, we must also recognise that we are not entirely passive in the face of them. The CHTs ‘Digital Well-Being Guidelines during the COVID-19 Pandemic’ has some effect on online behaviour, as well as the online virtues that Vallor and Harris advocate. The problem with these two approaches is that they seem to imply that the entire burden of responsibility for digital well-being should be shouldered by individual users. In our examination of the PT and situationist literature, we have seen that this view is manifestly unfair. Our behaviour is strongly influenced by the design of online environments and the PTs that populate them, so our digital well-being cannot be something for which we are solely responsible. Again, we must walk the line between admitting that we are individually responsible for our digital well-being to some extent, while recognising that this responsibility has limits.

Fourth, acknowledging that there is a role for individual responsibility in digital well-being, helps avoid charges of paternalism. Designing online environments in a way that seamlessly led to digital well-being, could be said to undermine the autonomy and self-determination of users. While it is not currently possible to design an online environment that consistently promotes digital well-being, doing so would put us in danger of being overly paternalistic. As noted above, benign paternalism is still paternalism, which is especially worrying if autonomy is a part of well-being itself. This suggests that a comprehensive approach to digital well-being must draw from multiple strategies. Making use of our autonomy (rule-based, character-based strategies) *and* strategies that recognise that this autonomy has strict limits (VSD and PTs) offers the best way of preserving self-determination while also reducing the burden of individual responsibility.

## Conclusion

This article has surveyed three strategies for digital well-being, and has evaluated their strengths and weaknesses. I have argued that, taken alone, none of these strategies provides a comprehensive approach to digital well-being because each strategy has problems. It is vital to balance a wide range of ethical issues when deciding on the practical ways to promote digital well-being in a post-COVID world in which an increasing dimensions of our lives are likely to be online. It is a delicate task to ensure that worries about paternalism are mitigated by digital well-being strategies that emphasise individual responsibility. Similarly, it is hard to ensure that individualised strategies can fully recognise the strength of PTs that operate in today’s online environments. Understanding how to maintain this balance under pandemic conditions (as well as once the crisis has passed) is the key challenge that a comprehensive theory of digital well-being must address.^19^

## Footnotes

1 While it is likely that COVID-19 will affect online behaviour for the foreseeable future, there are already signs that many users are deeply unsatisfied with the widespread use of online technologies, especially in education. Hasnan Baber’s recent study suggests that traditional teaching methods ‘positively influence […] student satisfaction’ in a way that online teaching does not (2020, p. 285).

2 V. Strauss (2020). *The Washington Post*. Retrieved 15 May 2020 from www.washingtonpost.com/education/2020/05/06/cuomo-questions-why-school-buildings-still-exist-says-new-york-will-work-with-bill-gates-reimagine-education/.

3 N. Klein (2020). *The Guardian*. Retrieved 15 May 2020 from www.theguardian.com/news/2020/may/naomi-klein-how-big-tech-plans-to-profit-from-coronavirus-pandemic.

4 Looking further ahead, the COVID-19 crisis may present us with a useful lens to perceive slower-burning ecological problems. Whether locked down owing to a global pandemic, or housebound because of a degraded ecological environment, the future circumstances of our lives may well mean that we are forced to spend increasingly large amounts of our lives online (Vallor 2016, p. 117).

5 Burr and Floridi define digital well-being as the ‘impact that digital technologies, such as social media, smartphones, and AI, have had on our well-being and our self-understanding of what it means to live a life that is *good for* us in an increasingly digital society.’ (Burr and Floridi 2020, p. 3; emphasis in the original).

6 Kick-back in user engagement in the Global North has been difficult to discern because of an overall rise of active online users in the Global South. But despite an overall increase in social media users, from 2010 onwards tech companies noticed that an increasingly sizable group of users were logging off (Arora 2019).

7 How to interpret these results is still subject to debate. We can, of course, simply explain them as showing that those who lead a more active life have more to post online about. In this case, posting in itself would be unconnected to living well, but would merely supervene on a deeper relationship between well-being and an eventful, active life, etc.

8 While there is no hard-and-fast distinction between intrinsic and extrinsic motivation, there does seem to be a discernible difference between a motivation that comes from the person, and that which come from the environment that they are in (or their self-governance framework).

9 ‘Digital Well-Being Guidelines during the COVID-19 Pandemic.’ (1) Feel Into It; (2) Be “Why's”; (3) All Screen-Time Is Not Equal; (4) Remember, Tech is a Trade; (5) Get Proactive; (6) Choose the "Right" Tech; (7) Protect Developing Brains; (8) Be Skeptical (2020). Retrieved 9 May 2020 from www.humanetech.com/digital-wellbeing-covid.

10 The CHT’s examples of such value trade-offs are informative: ‘For example, with Google Maps we’re trading our location data for navigational convenience, and most of us are comfortable with that trade. Other times it takes a lot of reflection to recognize the deeper trade-offs. For example, we may be trading our time and peace of mind for seeing what others are posting, the trade is more than just being exposed to advertising. We also might be trading spending time on something that’s harder to do (like exercise) for being entertained.’ [Accessed 1st May 2020.].

11 Although autonomy has competing meanings in the literature, in what follows I use the term to mean a strong sense that a person has decision-making capacity and volition over their personal choice of behaviour.

12 Snapchat’s ‘streak’ function provides an instructive example of gamification. Here users are required to ‘snap’ with their contacts on a daily basis in order to maintain a ‘streak’, which symbolizes the friendship bond between users. The desire to maintain this bond creates a powerful motivation for users to use the app on a daily basis (Vaterlausa 2016).

13 While the burden of responsibility for one’s character is often thought to be less clear cut than the responsibility for not obeying rules, there are times when we can be blamed for having acquired vices rather than virtues.

14 This could be for two reasons: (1) character may be not robust enough to consistently guide conduct, or (2) character might only be robust in offline contexts, but the strength of PTs are too formidable. While Doris (2002) and Harman (1999) cast doubt on the robustness of character, empirically informed work on PTs suggest they may be even more powerful (Frank 2020; Lanzig 2019, 2018; Sullivan and Reiner 2019).

15 Doris’ empirical studies include a famous experiment on trainee clergymen who were on their way to present a sermon on the Good Samaritan. In this study, trainees were told they only had minutes to spare to get to the lecture. On the way to the lecture hall, they were waylaid by a stooge masquerading as a mendicant who began loudly pleading for assistance. Only 10% of the clergymen stopped to help. (Darley and Batson 1973, p. 100).

16 Studies such as Carrier et al. suggest that the reason empathy decreases online is because of the *physical detachment* that users of social media technologies typically have in relation to the others that they are communicating with (2015, p. 48).

17 See also Cocking and van den Hoven’s focused discussion of moral character and situationism (2019, pp. 120–4).

18 For van de Poel, the epistemic problem can be addressed by a better understanding of the unpredictable ways that technology affects a range of stakeholders (often a greater of number of individuals than the mere ‘users’ of the technology), whereas the aggregation problem can potentially be surmounted by paying greater attention to how some prudential values can lead to incompatibility and various kinds of moral conflicts.

19 I would like to thank two anonymous reviewers for their constructive and insightful comments.
